# A retinal model of cerebral malaria

**DOI:** 10.1038/s41598-019-39143-z

**Published:** 2019-03-05

**Authors:** François Paquet-Durand, Susanne C. Beck, Soumyaparna Das, Gesine Huber, Le Chang, Timm Schubert, Naoyuki Tanimoto, Marina Garcia-Garrido, Regine Mühlfriedel, Sylvia Bolz, Wolfgang Hoffmann, Ulrich Schraermeyer, Benjamin Mordmüller, Mathias W. Seeliger

**Affiliations:** 1 Cell Death Mechanisms Lab, Institute for Ophthalmic Research, University of Tübingen, Tübingen, Germany; 2 Division of Ocular Neurodegeneration, Institute for Ophthalmic Research, University of Tübingen, Tübingen, Germany; 3 Institute for Ophthalmic Research, University of Tübingen, Tübingen, Germany; 4 Centre for Integrative Neuroscience (CIN), University of Tübingen, Tübingen, Germany; 5 Institute of Tropical Medicine, University of Tübingen, Tübingen, Germany; 6 Experimental Vitreoretinal Surgery, Institute for Ophthalmic Research, University of Tübingen, Tübingen, Germany

## Abstract

Malaria is a causative factor in about 500.000 deaths each year world-wide. Cerebral malaria is a particularly severe complication of this disease and thus associated with an exceedingly high mortality. Malaria retinopathy is an ocular manifestation often associated with cerebral malaria, and presumably shares a substantial part of its pathophysiology. Here, we describe that indeed murine malaria retinopathy reproduced the main hallmarks of the corresponding human disease. In the living animal, we were able to follow the circulation and cellular localization of malaria parasites transgenically labelled with GFP via non-invasive *in vivo* retinal imaging. We found that malaria parasites cross the blood-retinal-barrier and infiltrate the neuroretina, concomitant with an extensive, irreversible, and long-lasting retinal neurodegeneration. Furthermore, anti-malarial treatment with dihydroartemisinin strongly diminished the load of circulating parasites but resolved the symptoms of the retinopathy only in part. In summary, we introduce here a novel preclinical model for human cerebral malaria that is much more directly accessible for studies into disease pathophysiology and development of novel treatment approaches. *In vivo* retinal imaging may furthermore serve as a valuable tool for the early diagnosis of the human disease.

## Introduction

Malaria is considered one of the most common and deadliest diseases that are afflicting mankind^1^. Cerebral malaria is a complication that is associated with a poor prognosis^2^ and can lead to irreversible sequelae, which can have a profound effect, particularly, on child development, although such sequelae often remain unnoticed^3,4^. The precise pathological processes and mechanisms leading to cerebral malaria are still unclear, hampering the development of efficacious treatments. Typically, cerebral malaria is accompanied by malaria retinopathy, which is considered as an early symptom for most forms of cerebral malaria^5,6^.

The retina is an integral part of the central nervous system and combines easy access with the possibility to directly visualize neurodegenerative processes *in vivo*, in a non-invasive fashion^7^. The retina is perfused by two vascular systems, the outer retinal vasculature or the choroid and the inner retinal vasculature. The neuroretina is shielded from blood-borne toxins or infectious agents by the blood-retinal-barrier (BRB). As for the vasculature, the BRB consists of two morphologically distinct parts, the outer retinal, choroidal BRB, which is formed by fenestrated endothelial cells in conjunction with retinal pigment epithelial cells (RPE), and the inner retinal BRB, which is formed by non-fenestrated endothelial cells in conjunction with Müller glial cells^8^. While the outer BRB is a retina specific tissue adaptation, the inner BRB is virtually identical to the blood-brain-barrier in other parts of the central nervous system^9,10^.

Mice can develop neurological signs following infection with certain mouse-pathogenic species of malaria parasites including *Plasmodium berghei*. The C57BL/6J mouse strain is particularly vulnerable to developing the symptoms of cerebral malaria, something that typically occurs within 8–10 days post-infection (DPI) and is usually fatal after 1–2 more days^11^. Even though the mouse is an often used model system for studies into the underlying pathophysiology as well as for drug candidate testing and treatment development^12^, malaria-induced murine retinopathy has not been studied in any detail^13^.

Here, we present the first systematic investigation on malaria induced retinopathy in the mouse using both *in vivo* and *ex vivo* techniques for a comprehensive longitudinal study of disease pathogenesis. Our results demonstrate that the ocular and retinal symptoms in the mouse model are reproducing the main hallmarks of human malaria retinopathy. Moreover, we provide strong *in vivo* and *ex vivo* evidence that malaria parasites cross the BRB and infiltrate the neuroretina. We also show that malaria parasites trigger extensive, irreversible and long-lasting retinal neurodegeneration, the symptoms of which may persist even after timely anti-malarial treatment. Finally, we suggest that *in vivo* imaging techniques routinely used in the clinic, namely confocal scanning laser ophthalmoscopy (SLO) and spectral domain (SD) optical coherence tomography (OCT), offer a significant detection advantage for malaria retinopathy and could thus constitute a valuable new tool for the early diagnosis of cerebral malaria in humans as well as for evaluation of effective therapeutic approaches *in vivo*.

## Materials and Methods

### Animals and parasites

C57BL/6J mice were obtained from Charles River (Germany), were housed under standard (12 h) white cyclic lighting and had free access to food and water. Only male animals were used in this study. All procedures involving animals were performed in accordance with the ARVO Statement for the use of animals in Ophthalmic and Vision Research. The study and the protocol (Registration No. T1/10) were approved by the competent local authority, Regierungspräsidium Tübingen, based on the assessment by the appointed regional ethics board, and according to the applicable German law on animal protection (Tierschutzgesetz). The *P*. *berghei* parasites (ANKA strain) used for the experiments, were kindly provided by A. Walliker, Institute of Cell, Animal, and Population Biology, University of Edinburgh, United Kingdom.

### Generation of GFP expressing *P. berghei* parasites and controlled infections

GFP expressing *P*. *berghei* were generated as previously described^14,15^. The transfection vector (pl0016) was kindly provided by The Leiden Malaria Research Group, LUMC, Leiden, Netherlands. Host animals were infected with frozen stocks, monitored until parasitaemia reached 1–5% and bled around midday, giving rise to mixed blood stage parasites with more than 80% rings and young trophozoites. Research for cerebral malaria typically uses sub-adult (*e*.*g*. three weeks old) C57BL/6J mice, producing a very severe disease phenotype that does not allow to follow the animals for prolonged periods of time. For our study, we used adult, male C57BL/6J mice (1–4 months old), which were infected intravenously (i.v.) with 1 Mio. parasites using standard protocols^16^. Previous experience indicated an onset of cerebral malaria in this age group after about 8 days post-infection (8DPI) with this infectious dose (data not shown). All infected mice were treated orally with pyrimethamine throughout the experiment to avoid parasite reversion to wild-type.

### Non-invasive diagnostics: retinal imaging (SLO/OCT) and electroretinography (ERG)

In a first group of five animals (male, one month old), infected with *P*. *berghei*, retinal changes were determined by confocal scanning laser ophthalmoscopy (SLO), spectral domain (SD) optical coherence tomography (OCT) and electroretinography (ERG) at 3DPI^17,18^, *i*.*e*. before mice showed any signs of neurological involvement, followed by a second measurement at the expected onset of cerebral malaria (6DPI). *In vivo* imaging observations were performed and confirmed by three independent examiners (SB, GH, MGG). On those days, parasitaemia was determined on Giemsa stained blood smears. The *in vivo* analyses were performed consecutively beginning with ERG recordings, followed by OCT and SLO imaging. The mice were anaesthetized using a combination of Ketamine (66.7 mg/kg body weight) and Xylazine (11.7 mg/kg body weight) and their pupils dilated^19,20^. ERGs were recorded binocularly according to previously described procedures^18^. Briefly, single flash ERG responses were obtained under scotopic (dark adapted overnight) and photopic (light adapted with a background illumination of 30 cd/m^2^ starting 10 min before recording) conditions. Single white-flash stimuli ranged from −4 to 1.5 log cd*s/m^2^ under scotopic and from −2 to 1.5 log cd*s/m^2^ under photopic conditions. Ten responses were averaged with inter-stimulus intervals of 5 s (for −4 to −0.5 log cd*s/m^2^) or 17 s (for 0 to 1.5 log cd*s/m^2^).

SLO imaging and angiography was performed with a Heidelberg retina Angiograph; (Heidelberg Engineering GmbH, Dossenheim, Germany) according to a previously published method^18^. For native fundus imaging laser wave lengths of 830 nm and 514 nm were used, fundus autofluorescence analysis (AF) was performed with 488 nm. For angiography, both fluorescein (FL) and the argon blue laser at 488 nm (barrier 500 nm), and indocyanine green (ICG) and the infrared laser with 795 nm (barrier 800 nm), were used.

Retinal layer morphology was visualized via OCT imaging with a SpectralisTM HRA + OCT (Heidelberg Engineering GmbH, Heidelberg, Germany), as reported previously^7,21^. This device features a superluminescent diode at 870 nm as low coherence light source. Scans are acquired at a speed of 40.000 scans per second, with each two-dimensional B-scan containing up to 1536 A-scans. The images were taken with the equipment set of 30° field of view, using Heidelberg Eye Explorer software (HEYEX version 5.3.3.0, Heidelberg, Germany). Resulting images were exported as 8-bit colour bitmap files and processed with CorelDraw X5 (Corel corporation, Ottawa, ON Canada).

Concerning the quantification of mouse SLO and OCT datasets, we note that there are marked differences to human retinal imaging where the much larger size of the human eye, the corresponding calibration of imaging equipment, and the availability of large training sets of images of human pathology largely facilitates quantification. For mouse imaging specifically, the following limitations apply:

- Since the mouse eye is very small it is only accessible to human-rated equipment (which we use) via the use of adaptor lenses that limit the observation angle to approx. 30°.
- The exact angle of observation is variable to about 10–15° and can only be estimated within this range. This means that also sizes and distances cannot be quantified precisely.
- Imaging of the retinal periphery in the mouse–where many of the malaria-induced aberrations occur – requires tilting the observation leading to further image distortion.
- Fluorescence intensities also vary with the illumination angle, thus only relative differences in fluorescence intensities can be evaluated.

### Curative treatment with dihydroartemisinin

To investigate reversibility of the observed retinal changes, a second group of six animals (male, four months old) were infected *i*.*v*. as described above and treated with the anti-malarial drug dihydroartemisinin (DHA). Pyrimethamine was administered orally via drinking water to avoid parasite reversion to wild-type (see above). *In vivo* measurements (OCT, SLO, ERG; as above) took place on 3DPI and 6DPI. Following the second measurement, all animals were treated with daily intraperitoneal injections of 30 mg/kg DHA from 6DPI to 18DPI, and were followed up with further measurements until 18DPI.

### Histology and TUNEL assay

Following the last measurement mice were sacrificed using CO_2_ asphyxiation, the eyes were enucleated and then either fixed in 4% paraformaldehyde (PFA) or not fixed and flash frozen on liquid N_2_ and then stored at −20 °C (*i*.*e*. unfixed). The eyes were cryoprotected in increasing concentrations of sucrose (10, 20, 30%). After embedding in cryomatrix (Tissue Tek®, Sakura Finetek, Zoeterwoude, The Netherlands), the eyes were sectioned in a Microm HM cryostat (12 µm sections). A haematoxylin-eosin staining was used for routine histology.

Detection of dying cells was performed as reported previously^22^, using the terminal deoxynucleotidyltransferase (TdT) dUTP nick end labelling (TUNEL) assay (Roche Diagnostics, Mannheim, Germany). Microscopy was performed on a Zeiss Imager Z1 Apotome Microscope; images were captured with Zeiss Axiovision 4.8 software (Zeiss, Wetzlar, Germany).

### Immunofluorescence

Retinal tissue sections were rehydrated in PBS and incubated at 4 °C over night in primary antibodies (Table 1). Primary antibodies were detected with Cy3-conjugated (Jackson ImmunoResearch Laboratories) or Alexa Fluor 488-conjugated secondary antibodies (Molecular Probes). Sections were embedded with Vectashield mounting medium with DAPI (Vector Laboratories) and viewed using a Zeiss Imager Z1 Microscope equipped with an Apotome deconvolution unit. For visualization of retinal blood vessels CF8 or Glut1 immunostaining was used.

**Table 1:** Antibodies used in the study.

Antibody, Host	Manufacturer	Order No.	Dilution
Anti-GFP, rabbit	Merck Millipore, Darmstadt, Germany	AB3080	1:400
Anti-complement factor 8 (CF8), mouse	DAKO (Agilent), Waldbronn, Germany	M0616	1:50
Anti-GFAP, mouse	Sigma-Aldrich, Taufkirchen, Germany	G-3893	1:400
Anti-glucose transporter 1 (Glut1, *SLC2A1*), rabbit	Merck Millipore, Darmstadt, Germany	07–1401	1:200
Anti-PAR (clone 10H), mouse	Acris Antibodies GmbH, Herford, Germany	SM1398	1:200

### Calpain *in situ* activity assay

The whole unfixed frozen eyes were embedded in Tissue Tek and cryosectioned at 12 μm. Unfixed retinal sections were incubated for 15 min in calpain reaction buffer (25 mM HEPES, 65 mM KCl, 2 mM MgCl_2_, 1.5 mM CaCl_2_, 2 mM DTT, pH 7.2)^23^. The fluorescent calpain substrate CMAC, t-BOC-Leu-Met (A6520, Life technologies, Darmstadt, Germany) was then added to the reaction buffer at a final concentration of 2 μM and sections plus reaction mixture were incubated in the dark for 2 h at 37 °C. The sections were washed twice for 10 min each in reaction buffer and then mounted with Vectashield (Vector, Burlingame, CA, USA). The activity assay generally labelled the cell membranes, while calpain-activity-positive cells additionally showed a bright labelling of the nucleus and perinuclear cytoplasm^24^.

### Quantification and statistics

For quantification of cells positive for a given marker (*i*.*e*. TUNEL, calpain activity, PAR), pictures were taken from three to five sagittal sections, within 5–10° eccentricity from the optic nerve, for at least four different animals for each experimental condition using Axiovision 4.8 and 20 x magnification. The average ONL areas used for quantification were equivalent ranging from approx. 20.000–35.000 µm^2^. Uninfected, healthy adult C57BL/6J mice were used as controls. The average surface area occupied by a photoreceptor cell (*i*.*e*. cell size) for each genotype was determined by counting DAPI-stained nuclei in 9 different areas (50 × 50 µm) of the retina^25^. The total number of photoreceptor cells in a given ONL area was estimated by dividing area by the average cell size. The number of positively labelled cells in the ONL was counted manually.

To quantify relative staining intensities in GFP and GFAP stained microscope images, we used histogram data captured by Zeiss ZEN 2.3 software (Zeiss) for each microscope image (3–8 images per specimen) to measure the pixels showing GFP or GFAP staining above threshold intensity. Individual pixel intensities ranged from 0–16384 (14 bit), an intensity above 2048 was considered as positive labelling. The number of GFAP positive pixels in infected retina at 6DPI was arbitrarily set to 100%, the numbers of GFAP positive pixels in uninfected retina and at 18DPI were expressed as a percentage of this. In the same way, GFP positive pixels were expressed as percentage of the 6DPI GFAP values.

Data were evaluated using Graph Pad Prism 7.03 software (GraphPad Software, La Jolla, CA, USA) and one-way ANOVA analysis with a Kruskal-Wallis test and Dunn’s multiple comparison post-test. Levels of significance were: ***p < 0.001; **p < 0.01; *p < 0.05. Data are presented in figures as a mean ± standard error of the mean (SEM).

## Results

**Figure 1:**
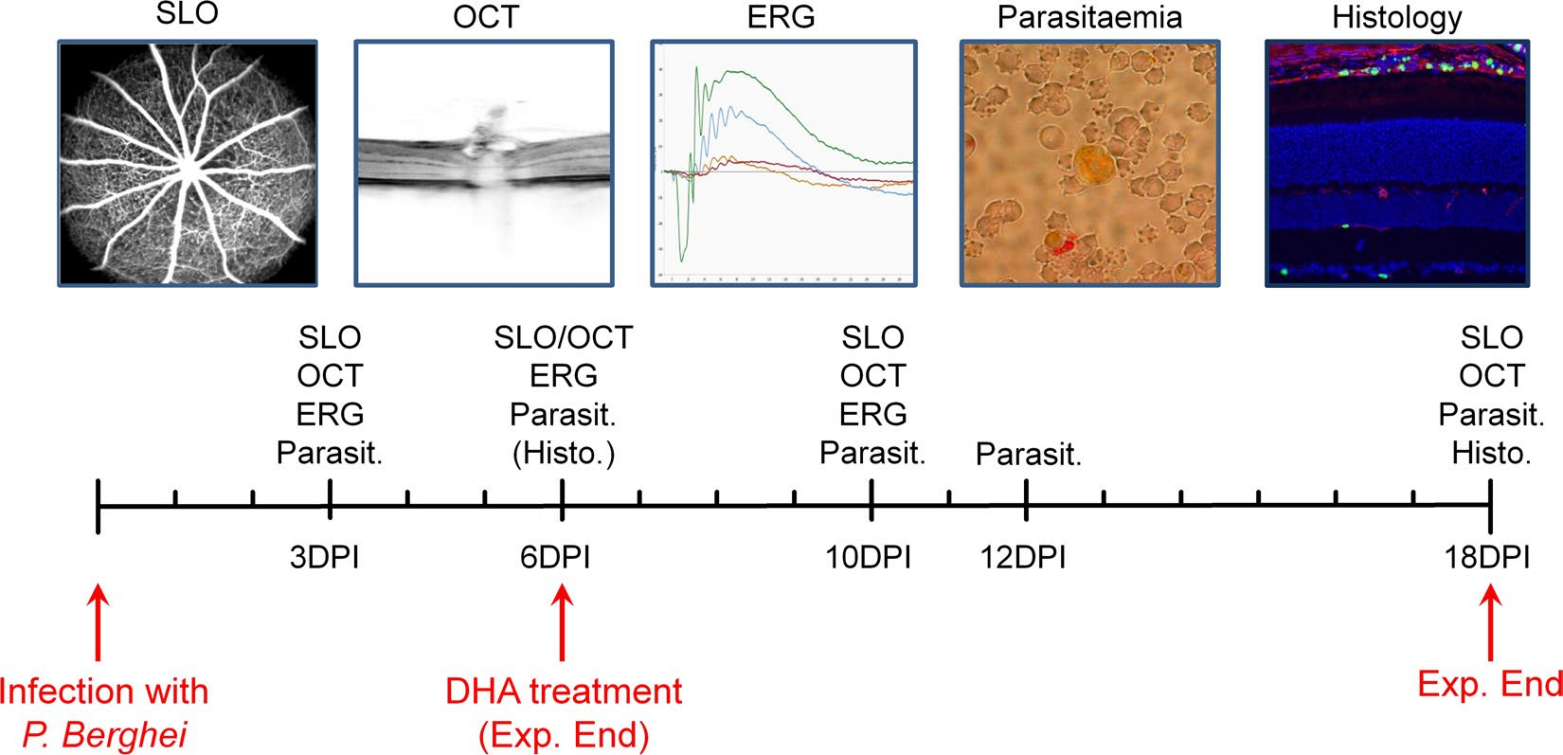
Experimental design. Animals were infected with *P*. *berghei* (1 Mio parasites i.v.) and examined on several days post-infection (DPI). Measurements included non-invasive, *in vivo* scanning laser ophthalmoscopy (SLO), optic coherence tomography (OCT), and electroretinography (ERG), as well as determination of parasitaemia and eventually histological workup and analysis. At 6DPI, after *in vivo* examination, the animals were treated with DHA and monitored for a further 12DPI until experiment end.

To induce malaria retinopathy animals were infected intravenously (i.v.) with 1 Mio parasites of the mouse pathogenic *P*. *berghei* ANKA strain, transgenically labelled with GFP^14^. At various time-points after infection (days post-infection, DPI) mice were examined using non-invasive morphological and functional ocular testing. Among the tests used were scanning laser ophthalmoscopy (SLO), optical coherence tomography (OCT), and electroretinography (ERG), as well as determination of parasitaemia. After 6DPI animals were treated with dihydroartemisinin (DHA), a standard anti-malarial drug and closely monitored for further 12 days until the end of the experiment (18DPI) and full histological work-up and analysis (Fig. 1).

### Direct, *in vivo* visualization of GFP expressing *P. berghei* in the retina

**Figure 2:**
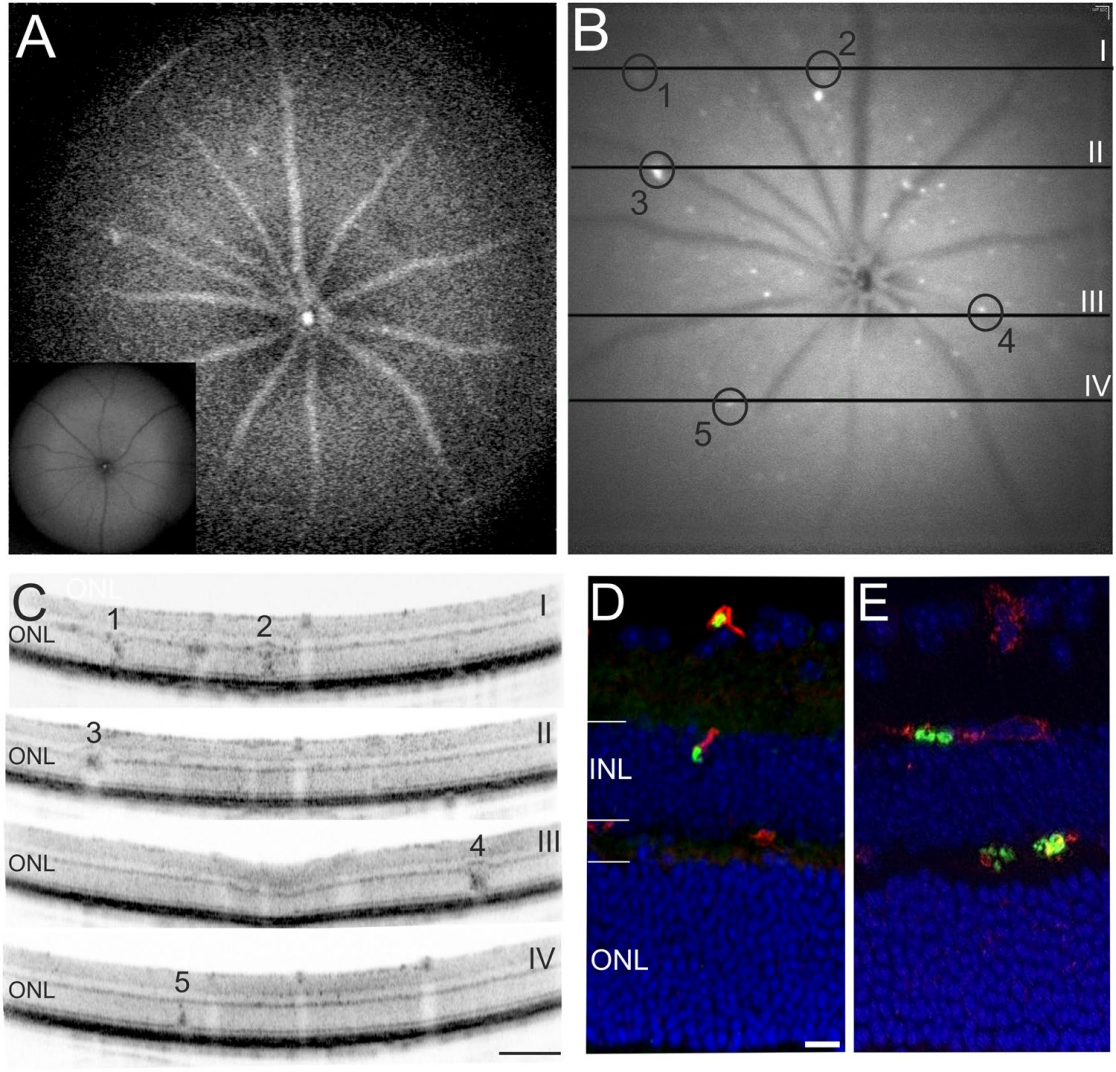
*In vivo* and *ex vivo* detection of GFP-labelled *P. berghei* in the retina. (**A**–**C**) *in vivo* imaging of a representative animal at 3DPI. (A) SLO AF mode (overview) visualized plasmodia flowing in the blood stream of the large retinal vessels and capillaries (inset: uninfected wild-type). (**B**) SLO AF mode with focus into deeper retinal layers detected clusters of GFP expressing parasites inside the retina. Regions of interest (ROI) are denoted by black circles. The lines depict the position of the OCT sections. (**C**) OCT sections with correspondingly numbered ROI visualizing sequestration of parasites. (**D**,**E**) Histological sections detected GFP expressing clusters of plasmodia (green) that colocalized with vascular CF8 staining (red). DAPI (blue) was used as nuclear counterstain. Scale bar in C = 200 µm; D = 20 µm.

The autofluorescence (AF) mode in SLO imaging allows for the *in vivo* detection of single cells expressing GFP^26^. In uninfected animals, SLO AF imaging reveals the normal grey fundus appearance with the dark overlying retinal vessels (Fig. 2A, inset). In contrast, AF imaging of infected animals revealed GFP expressing plasmodia flowing in the blood stream as bright signals within the large retinal vessels (Fig. 2A; Supplemental Video 1). SLO imaging focusing into deeper retinal layers detected GFP signals that were unevenly scattered over the fundus and were not noticeably moving with the blood stream (Fig. 2B).

Immobile parasites colocalized to retinal layer alterations that were visualized by combined *in vivo* SLO and OCT imaging (compare numbered circles in Fig. 2B,C). The normal retinal layer morphology found in control animals (Fig. 1, OCT) was preserved in most areas of the retina in experimental animals (Fig. 2C). In contrast, the presence of plasmodia resulted in numerous local alterations that were distributed over the entire fundus and the retinal cross sections (Fig. 2C, 1–5; Supplemental Video 2). At present, it is unclear whether the variable size of the observed alterations relates to differing degrees of parasite sequestration within retinal vessels, as described for patients suffering from malaria retinopathy^27,28^.

**Figure 3:**
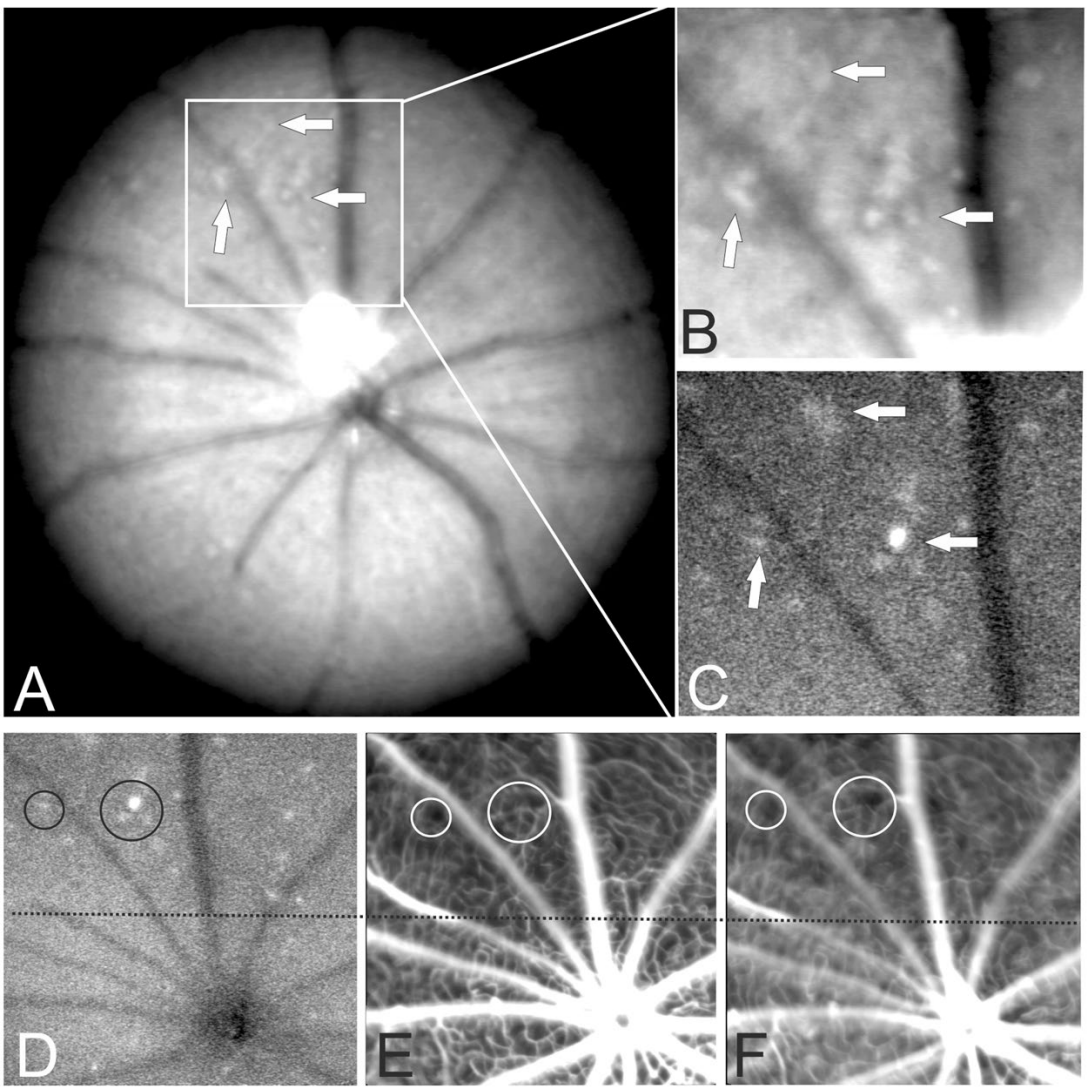
*In vivo* SLO imaging directly correlates altered retinal morphology with presence of plasmodia. (**A**) Native fundus imaging at 3DPI. (**B**) Magnification. (**C**,**D**) AF mode detecting GFP expressing plasmodia. (**E**) Fluorescein angiography, (**F**) Indocyanine green angiography. Areas of retinal whitening (arrows in **A**, 20° fundus overview and **B**, magnification) could directly be correlated to the presence of GFP expressing plasmodia (arrows in **C**). Several sites of capillary non-perfusion (CNP) were detected by angiography (**E**, fluorescein and **F**, indocyanine green) that could directly be correlated to the plasmodia (examples are denoted by circles in **D**–**F**) and the areas of retinal whitening (**A**,**B**). The numerous sites of CNP accumulated to a large area of reduced tissue perfusion affecting a considerable part of the visible fundus (above the dotted line in **D**–**F**).

**Figure 4:**
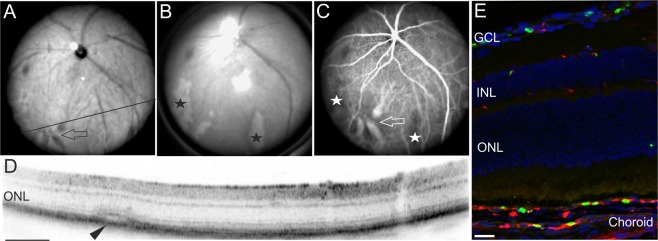
Severe symptoms of malaria retinopathy affecting large vessels. Examination of a representative infected mouse at 6DPI. (**A**) SLO native imaging with IR mode depicting the position of the respective OCT scan (**D**). (**B**) SLO native imaging in RF mode revealed large areas of retinal whitening (asterisks) that could be correlated to areas of non-perfusion (**C**, asterisks), vascular dilation in native imaging (**A**, arrow) and in ICG angiography (**C**, arrow), as well as with fluid accumulation seen in OCT imaging (**D**, arrowhead). (**E**) Histological sections detected sequestration of parasites in choroidal vessels. GFP expressing plasmodia (green), vascular CF8 staining (red). Scale bar in D = 200 µm; E = 40 µm.

**Figure 5:**
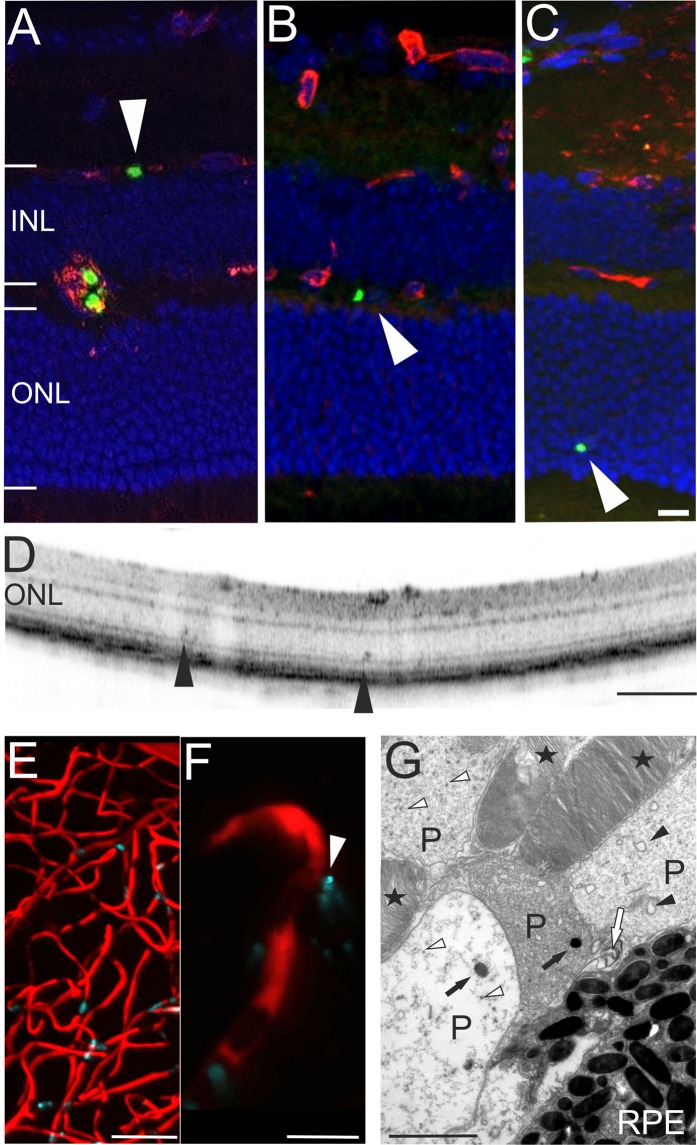
Evidence for extravascular parasites. (**A**–**C**) Histological sections revealed parasites (green) that did not colocalize to the vascular CF8 staining (red). In both, OCT imaging (**D**, arrowheads) and *ex vivo* histology (**C**, arrowhead) plasmodia were detected in the avascular outer retina. Two-photon microscopy analysis (**E**, overview) also revealed extravascular parasites (**F**, magnification). (**G**) In electron microscopy, plasmodia (**P**) displacing the photoreceptor outer segments (asterisks) could be identified based on membrane bound bodies (black arrow heads), food vacuoles with pigment (black arrows), pigment granules (white arrow heads), and the inner membrane complex or plastid of the parasite (white arrow). RPE, retinal pigment epithelium; scale bars: C = 20 µm, D = 200 µm, E = 50 µm, F = 10 µm, G = 2 µm.

These findings were corroborated by *ex vivo* immunohistology where clusters of plasmodia were also detected within the retinal vasculature (Fig. 2D,E). Comparable to the pathology in human malaria retinopathy^27^, also in the *P*. *berghei* ANKA mouse model the parasitized blood vessels presented a very different appearance, which depended directly on the degree of sequestration (Fig. 2C–E). In both OCT imaging and histological sections, higher (Fig. 2C, region of interest (ROI) #3, #4; Fig. 2E) and lower (Fig. 2C, ROI #1, #2, #5; Fig. 2D) degrees of sequestration were clearly distinguished.

### Pathological alterations in mouse retina resemble human malaria retinopathy

In human cerebral malaria and malaria retinopathy, sequestration is considered as a main pathophysiological mechanism of plasmodial infection. During infection, parasitized erythrocytes adhere to the vascular endothelium, which lead to narrowed and finally blocked vessels resulting in impaired perfusion and local tissue hypoxia. In human malaria retinopathy, the zones of capillary non-perfusion (CNP) and perfusion abnormalities were correlated to areas of retinal whitening^28,29^. In patients presenting with malaria retinopathy, the size of CNP with retinal whitening varied considerably from small spot-like zones to large affected areas; moreover, also the location varied from close to the centre to the retinal periphery^29^.

In infected mice, we detected pale, opaque areas (arrows in Fig. 3A,B) in the central retina, and also in the retinal periphery (Fig. 4A–C), which resembled retinal opacifications or areas of retinal whitening reported in human patients^29^. Using GFP expressing plasmodia and SLO *in vivo* imaging, we were able to directly colocalize the areas of retinal whitening (Fig. 3B) with the presence of the parasites (Fig. 3C).

The application of different detection wave lengths in SLO imaging allows the visualization of different entities at the exact same location within the retina. Thus, native fundus imaging of infected animals revealed white opaque areas (Fig. 3A, overview, Fig. 3B, magnification) which were comparable to the appearance of small areas of retinal whitening in human malaria retinopathy^29^ and, with switching to the AF imaging mode, could be directly correlated to the bright signals of GFP expression and thus to malaria parasites (Fig. 3C,D). Moreover, both, the white areas in native imaging, shown in magnification Fig. 2B, and the bright GFP signals, shown in Fig. 2C, were exactly at the same relative localization in relation to the two large dark appearing retinal vessels which are shown in both imaging modes (Fig. 2B,C). This again stresses the *in vivo* colocalization of parasites and sites of retinal whitening.

Moreover, with angiography, sites of capillary non-perfusion (CNP) and retinal perfusion abnormalities were observed (Fig. 3E,F denoted by corresponding circles). Similar to the observations in human malaria retinopathy, in the mouse model, the areas of retinal whitening (Fig. 3B arrows) and impaired blood flow (Fig. 3E,F circles) were also topographically matched. Moreover, we could directly colocalize the presence of parasites to these fundus alterations (Fig. 3C arrows, D circles).

To quantify the degree of colocalization between retinal whitening, GFP-positive parasites, and areas of low perfusion as assessed in angiography, we counted the spots showing fundus changes in SLO images. We took these spots as reference (=100%) and found that 97.4% (±2.6 STD) of these were also GFP positive. When comparing fundus changes to dark, non-perfused areas seen in angiography, we found an overlap in 85.9% (±1.6) of cases.

### Longitudinal studies reveal long-lasting effects of murine malaria retinopathy

Small retinal opacifications were observed at 3DPI as first signs of malaria retinopathy (Fig. 3). Three days later, at 6DPI, more severe symptoms had developed. Large areas of retinal whitening were visible (Fig. 4B, asterisks) that matched large regions of CNP (Fig. 4C asterisks). Moreover, SLO angiography detected congestion of choroidal vessels (Fig. 4A,C arrows). The impaired outflow from the occluded vessels resulted in considerable vessel dilation (Fig. 4A,C arrows) and fluid accumulation as shown by OCT imaging (Fig. 4D, arrowhead). These *in vivo* observations were corroborated by histological sections that revealed sequestration of parasitized red blood cells in the choroidal vasculature (Fig. 4E). Thus, similar to the symptoms of human malaria retinopathy^27,28,30^, this demonstrated sequestration and vascular congestion in large vessels likely resulting in ischemic areas of the peripheral retina (Fig. 4).

### Electroretinography does not show significant impairment of retinal function

Already at 3DPI *in vivo* imaging revealed retinal whitening and perfusion abnormalities (Fig. 3) and at 6DPI full malaria retinopathy was evident (Fig. 4). To assess whether these local alterations also affected the function of the neuroretina, functional studies based on full-field electroretinography (ERG) were performed. Measurement of dark adapted (scotopic), rod-dominated responses, and light adapted (photopic), cone-mediated responses, revealed no significant differences. Under both, scotopic (Fig. S1, left) and photopic (Fig. S1, right) conditions, the b-wave amplitudes were in the same range at 3, 6, and 10DPI (*i*.*e*. 4 days of DHA treatment). Thus, the local retinal aberrations induced by plasmodia infection had not yet produced detectable changes in ERG responses.

### *In vivo* and *ex vivo* data indicate parasite extravasation and neuroretina infiltration

*In vivo* imaging as well as in *ex vivo* histology, detected parasites in large retinal vessels (Fig. 2A), in all three capillary layers (Fig. 2) and in the choroidal vasculature (Fig. 4D,E). However, in histological sections some GFP labelled plasmodia were not colocalized to the CF8 signal of the vasculature (Fig. 5A–C arrowheads). Also, in two-photon microscopy of retinal capillaries (Fig. 5E, overview), GFP positive plasmodia were located outside the vascular lumen (Fig. 5F, magnification, arrowhead). Moreover, parasites could be found in the avascular region of the ONL (Fig. 5C). *In vivo* OCT imaging detected plasmodia deep in the ONL, just within the photoreceptor layer (Fig. 5D arrowheads). This result was clearly corroborated by electron microscopy that located plasmodia (*i*.*e*. trophozoites in various stages^31–33^) next to the outer segments of the photoreceptors (Fig. 5G). Altogether, these observations strongly suggested that some parasites had traversed the BRB and migrated into the surrounding neuronal tissue. This implies an at least local breakdown of the integrity of the retinal vasculature and thus of the BRB, and resembles the breakdown of the BBB, which is suggested as a mechanism in development of cerebral malaria^12,34^.

### *Plasmodium* infection causes cell death in the neuroretina

**Figure 6:**
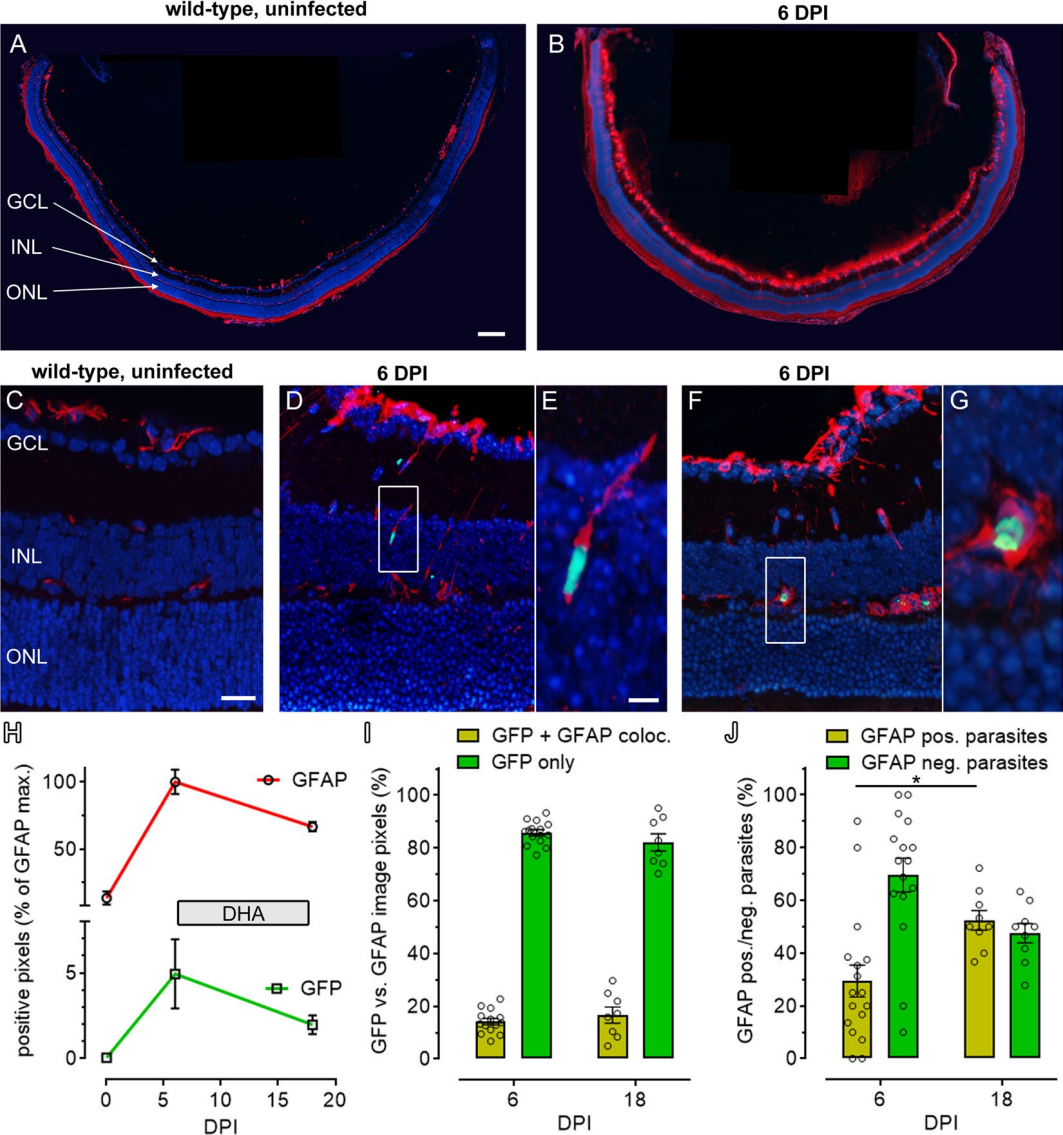
Müller glial cell activation indicates neuronal cell death. Uninfected wild-type mice presented with GFAP immunoreactivity (red) in the end feet of Müller glial cells (**A**,**C**), In contrast, at 6DPI, malaria infected animals displayed a marked increase of GFAP expression, leading to a labelling of entire Müller cells spanning the whole retina from GCL to ONL (**B**, **D**–**G**). Areas of strong GFAP immunoreactivity correlated with presence of *P*. *berghei* parasites (green) and in some cases parasites appeared localized within glial cells. GFAP and GFP labelling within the retina increased markedly from 0 to 6DPI and decreased with DHA treatment until 18DPI (**H**). A comparison of GFP and GFAP co-labelled image pixels did not reveal clear changes from 6DPI to 18 DPI (**I**). However, counting of GFP-positive parasites that were associated with GFAP-positive Müller cells showed a significant increase of parasites within Müller cells (*i*.*e*. GFAP positive parasites) from 6DPI to 18DPI (**J**). Images in (**E**,**G**) are close-ups of (**D**,**F**) DAPI (blue) was used as nuclear counterstain; scale bars: A = 200 µm, C = 25 µm, E = 5 µm. n (6DPI) = 14–17 observations from 4 animals, n (18DPI) = 8–9/2; statistical comparison: two-tailed t-test; *p < 0.05.

The infiltration of malaria parasites across the BRB into the neuroretina prompted us to search for signs of a potential neurodegeneration. We first studied the expression of glial fibrillary acidic protein (GFAP), which, in the healthy retina, is expressed at a low level in Müller glial cells. However, when Müller cells meet dying neurons, GFAP is strongly up-regulated, making it a very sensitive, general marker for retinal neurodegeneration^35^. In uninfected control animals, retinal GFAP immunoreactivity was almost absent and evident only in the cells end feet, next to the ganglion cell layer (GCL) (Fig. 6A,C). In infected animals, at 6DPI, strong GFAP expression was found throughout the retina (Fig. 6B). In uninfected animals, GFAP-labelling intensity was at 13.5% (±6.9 STD) of the values reached in infected animals at 6DPI (100%). Remarkably, some Müller glia cells were infected with parasites, further confirming that the parasites had crossed the BRB into the neuroretina (Fig. 6D–G).

At 6DPI, infected animals received a treatment with DHA to reduce parasite load. Within four days the treatment essentially cleared the blood stream of detectable parasites (Fig. S2A). At the same time, malaria infection also resulted in a marked loss of body weight in experimental animals in line with previous studies^34,36^. DHA treatment halted further weight loss until the end of the experiment (Fig. S2B). Apart from decreased weight, animals treated for 12 days with DHA appeared normal and showed no obvious signs of disease at 18DPI. Compared to the 6DPI values, GFAP immunoreactivity in 18DPI retinal tissue sections was reduced to 69.2% (±6.4 STD), but still elevated when compared to control. Relative to 6DPI GFAP-positive labelling, 4.9% (±3.5) of pixels were positive for GFP at 6DPI. This value dropped to 2.6% (±1.6) at 18DPI (Fig. 6H). Colocalization between GFP and GFAP was relatively low, with 14.2% (±4.5) and 16.5% (±8.6) of all GFP positive pixels also positive for GFAP at 6DPI and 18DPI, respectively (Fig. 6I). Indeed, rather than colocalizing, in many cases the GFP-positive parasites appeared to be displacing the GFAP label inside Müller glial cells.

To more precisely assess how many parasites were inside Müller glia cells, the intraretinal GFP-positive parasites that were associated with Müller cells, and those that were not, were counted. Counting showed a significant increase of parasites in Müller cells from 6DPI (29.4% ± 6.1) to 18DPI (52.5 ± 3.7; p < 0.05), suggesting Müller cells may have shielded parasites from the DHA treatment (Fig. 6J).

**Figure 7:**
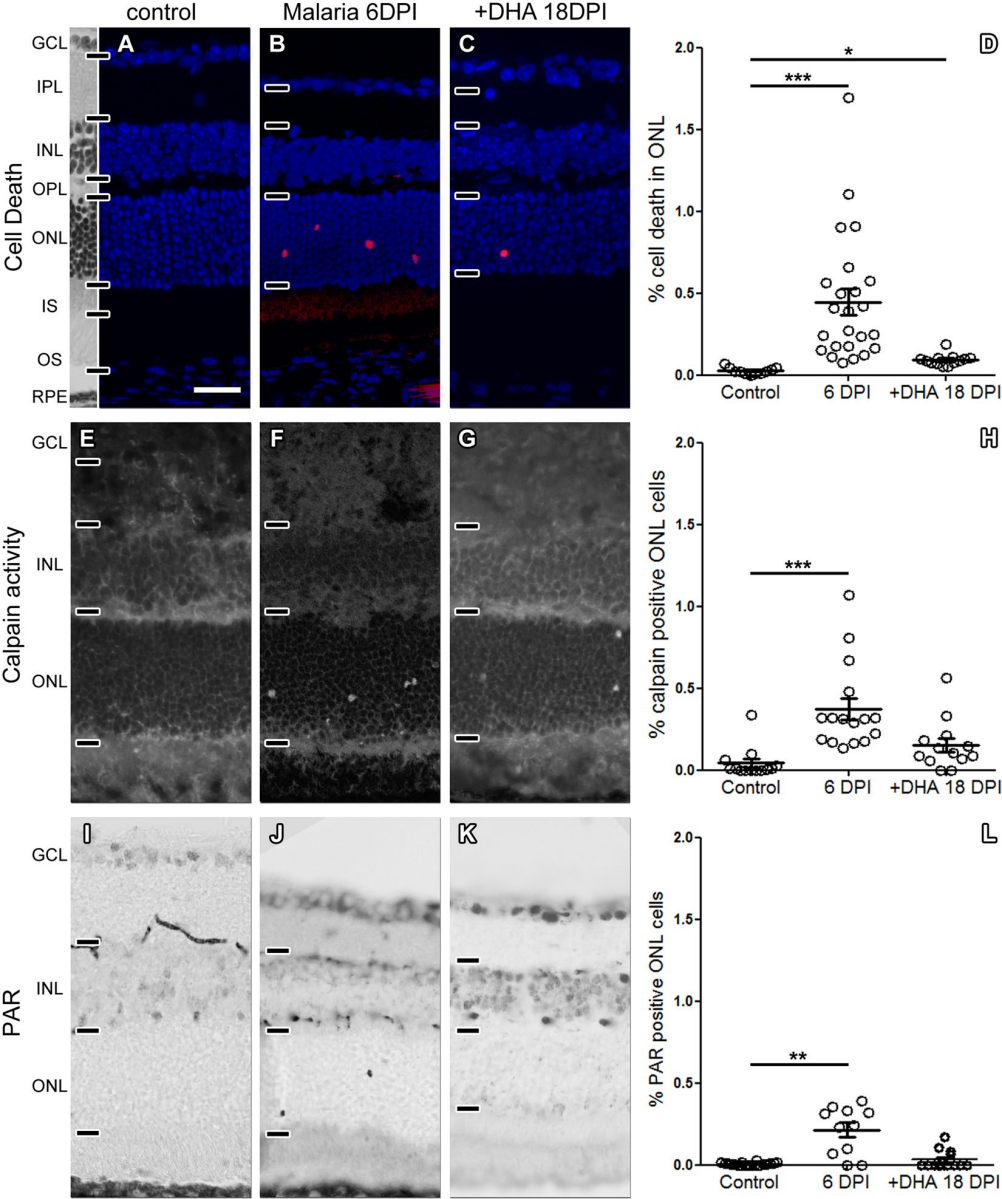
Malaria causes retinal neurodegeneration. (**A**,**B**) When compared to control, at 6DPI, the outer nuclear layer (ONL) of *P*. *berghei* infected mice presented with a significantly increased number of dying, TUNEL positive cells (red). (**C**) At 18DPI, after 12 days of DHA treatment, ONL cell death was reduced, but still significantly higher than in control. (**D**) Quantification for (**A**–**C**). (**E**–**L**) Similarly, calpain activity and cellular accumulation of PAR were increased at 6DPI and brought back down to almost pre-infection levels by DHA treatment. Scale bar in A = 50 µm; n (control) = 11 observations from 6 animals, n (6DPI) = 24/5; n (18DPI) = 13/4; statistical comparisons: one-way ANOVA with Dunn’s post-test, ***p < 0.001; **p < 0.01; *p < 0.05.

Since GFAP labelling strongly suggested a malaria induced retinal neurodegeneration, we further investigated this using the TUNEL assay^37^ to detect dying cells. At 6DPI a significant elevation of cell death was found in the outer nuclear layer (ONL; *i*.*e*. the photoreceptor layer), when compared to uninfected control retina. While the DHA treatment, starting at 6DPI, brought the number of dying ONL cells down to almost control levels by 18DPI (Fig. 7A–D), ONL cell death at that age was still significantly higher than in uninfected control animals. Occasionally, increased TUNEL positive cells were also seen in the inner nuclear layer (INL) and ganglion cell layer (GCL) (not shown).

To gain further insight into the mechanisms of cell death triggered by malaria infection, we tested for several markers characteristic for ONL cell death. This included the activation of Ca^2+^-activated calpain-type proteases^24^, where we found a significant increase in the numbers of ONL cells showing excessive calpain activity. These numbers were reduced to nearly control levels after DHA treatment at 18DPI (Fig. 7E–H). Similarly, a significant accumulation of poly-ADP-ribose (PAR) in ONL cells, previously shown to be connected to retinal cell death^22^, was observed in infected tissues, a phenomenon that was returned to control by DHA at 18DPI (Fig. 7I–L). Curiously, caspase-3, a protease key to the execution of apoptosis^38^, was not activated in retinas from malaria infected animals, suggesting a non-apoptotic mode of cell death (Fig. S3).

Taken together, these results indicated a long-lasting neurodegenerative effect of malaria infection on the retina, an effect that was not fully reverted even 12 days after the start of DHA treatment.

### Treatment with DHA only partly reverses disease phenotype

**Figure 8:**
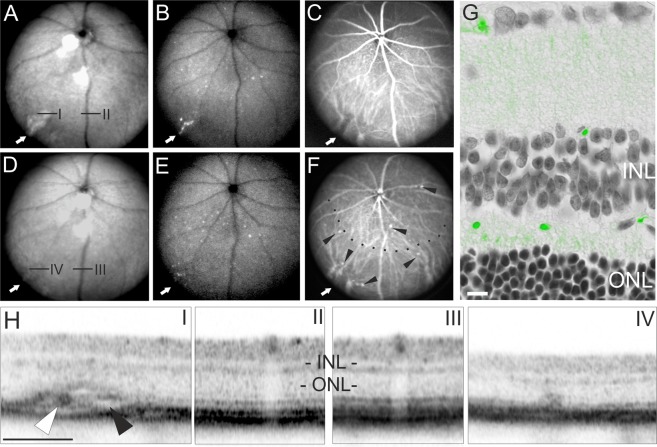
Longitudinal analysis of malaria retinopathy during treatment with DHA. (**A**–**F**) SLO imaging: (**A**,**D**) in RF mode, (**B**,**E**) GFP detection in AF mode, (**C**,**F**) ICG angiography. ROIs (I–IV); (**G**) histological section. (**H**) OCT sections corresponding to ROIs in the SLO images. ROIs I, IV show infected areas, while ROIs II, III show unaffected regions with healthy fundus appearance in SLO and normal retinal layering as assessed by OCT. (**A**–**C**, H I and II) show the analysis at 10DPI (*i*.*e*. after 4 days of DHA treatment). (**D**–**G**, H III and IV) show the 18DPI time point (12 days of treatment). Scale bars in G = 20 µm; H = 200 µm.

The course of malaria disease may be very different from one patient to another, including variations in parasitaemia, time course, and severity of disease^27^. This was also reflected in the *P*. *berghei* ANKA mouse model. For example, in one animal severe malaria retinopathy had already developed at day 6DPI (Fig. 4), whereas in another mouse, at the same time point, fundus alterations were less obvious (data not shown). However, by 10DPI severe malaria retinopathy had developed in all infected animals, including also in this mouse (Fig. 8). Retinal whitening (Fig. 8A arrow) was observed, which was tightly correlated to the presence of plasmodia (Fig. 8B arrow) and to occluded and dilated vessels in the choroidal vasculature (Fig. 8C arrow); OCT imaging revealed sequestered parasitized red blood cells (Fig. 8H,I white arrowhead) and fluid accumulation (Fig. 8G,I black arrowhead) within the affected choroidal vessel. To analyse the overall retinal layer morphology, a corresponding OCT image captured within the area of retinal whitening (denoted I in Fig. 8A,H) was directly compared to the adjacent unaffected retinal region (denoted II in Fig. 8A,H).

Notably, the symptoms of malaria retinopathy were not fully reverted by the DHA treatment and in part progressed even further. While at 18DPI the area of retinal whitening (Fig. 8D arrow) was apparently reduced in native SLO imaging, the GFP signals from the parasites were still detected in SLO AF imaging (Fig. 8E) and confirmed in histological sections (Fig. 8G). In ICG angiography the parasitized choroidal vessels still appeared enlarged and dilated (Fig. 8F, arrow). Moreover, further vascular alterations had developed (Fig. 8F, arrowheads) that were reminiscent of small aneurysm-like dilations (Fig. 8F, arrowheads), and a large area of low perfusion was now evident (Fig. 8F, below dotted line). On the other hand, in OCT imaging of the primary vascular alteration (Fig. 8H I) showed a regression after 12 days of treatment (Fig. 8H IV). However, analysis of the retinal layer morphology revealed a considerable reduction of the retinal thickness in the affected area whereas an adjacent unaffected retinal region did not show layer alterations (Fig. 8H; *cf*. III *vs*. IV).

Taken together, this data suggested that the loss of outer retinal thickness was localized to relatively small areas of the peripheral retina and may have been caused, for instance, by peripheral blood vessel occlusion (*i*.*e*. hypoxia), direct parasite interactions, or inflammatory processes. The data from the various *in vivo* and *ex vivo* retinal investigations is summarized in Table 2. Remarkably, this semi-quantitative overview highlights the value of OCT examinations, as a method for the *in vivo*, non-invasive, and early detection of malaria induced structural changes to the retina.

**Table 2:** Numbers of animals showing marked changes at different *in vivo* examinations.

Method/Exam	3DPI	6DPI	10DPI	18DPI
Parasitaemia in blood stream (also: GFP-labelled parasites moving in blood stream as observed in SLO; *cf*. Suppl. Fig. 2A)	11/11 100%	11/11 100%	1/6 17%	0/4 0%
OCT: Localized retinal layer alterations (at least 5 alterations, in at least 3 OCT scans)	11/11 100%	11/11 100%	6/6 100%	4/4 100%
SLO: Immobile GFP-labelled parasites (at least 10 GFP-labelled spots in visible fundus)	11/11 100%	11/11 100%	6/6 100%	4/4 100%
Histology (*ex vivo*): GFP detection, GFAP immunoreactivity (*cf*. Figs 5 and 6)	—	5/5 100%	—	4/4 100%

Animals were investigated at four consecutive points in time after infection with malaria (days post-infection; DPI). Data is expressed as the number of animals positive for a certain marker *vs*. the total number of animals examined, or as percentage. After the 6DPI exam, five animals were sacrificed, while six animals received DHA anti-malarial treatment and were investigated for a further 12 days.

## Discussion

The pathophysiological processes causing malaria retinopathy are not known even though it is hypothesized that they resemble the processes leading to cerebral malaria^39^. Here, we provide evidence that murine malaria retinopathy resembles human malaria retinopathy macroscopically and is associated with both changes of retinal vasculature and neurodegenerative processes. In this context, we also uncovered evidence for extravasation of malaria parasites and their migration into the neuroretina, which may explain at least some of the symptoms of cerebral malaria.

### Direct, non-invasive detection of malaria parasites in the living central nervous system

Among the techniques available for non-invasive *in vivo* retinal investigations are optical coherence tomography (OCT) for structural information, scanning laser ophthalmoscopy (SLO) for fundus analyses, angiography and (auto)fluorescent markers, and electroretinography (ERG) for functional analysis^7,40^. OCT and SLO techniques typically allow resolving features in the 7–8 µm range^41^ and allow for longitudinal follow-up studies. Previously, luciferase labelled *P*. *berghei* was used for detection of malaria infection in live animals and in explanted tissues and organs^42^. The resolution of this methodology was, however, relatively low (≈100 µM) and did not allow for the detection of individual cells. Higher resolution live imaging was afforded by an invasive confocal technique using an intracranial window in mice infected with GFP-labelled *P*. *berghei*^43^. Still, a major problem in malaria retinopathy research was to demonstrate a direct *in vivo* correlation between affected areas in retinal fundus imaging and the localization of parasites. Using GFP expressing *P*. *berghei* ANKA in the mouse^14^, we could effectively associate plasmodia to the pathological tissue alterations produced by the infection. To our knowledge, this is the first time that interaction of live plasmodia with a pathophysiologically relevant host tissue was monitored during the acute phase of malaria infection, using non-invasive, high-resolution *in vivo* imaging techniques.

The symptoms of murine malaria retinopathy observed in our study matched to large extent with corresponding human observations. For instance, retinal whitening^27^, peripheral vascular alterations^29^, blood vessel occlusion^44^, and GFAP immunoreactivity^28,30^ were all observed in both our mouse model and in human malaria retinopathy. Thus, our mouse model appears to be a close approximation of the human pathology, even though there are certain limitations when it comes to *in vivo* mouse eye imaging and human imaging (*cf*. Materials and Methods). However, an important difference to the human situation is the apparent absence of haemorrhages, which in our mouse model may be due to the fact that we could not investigate the very final stages of malaria retinopathy because of animal welfare concerns.

Previous studies suggested the use of funduscopy and retinal imaging to investigate malaria pathogenesis and in particular to allow for an early diagnosis of cerebral malaria before severe symptoms such as coma and convulsions set in (reviewed in^45^). Using *in vivo* imaging, we detected fundus alterations and retinal vascular abnormalities already at 3DPI. In the mouse, this corresponds to a time-point approximately five days before symptoms of cerebral malaria manifest themselves^11,43^, translating into a significant extension of the treatment window. Similarly, SLO angiography found very clear indications of localized low or no retinal perfusion at 3DPI. With progression of the disease further retinal alterations developed that could clearly be identified by OCT and SLO and were even more evident at 6DPI (*i*.*e*. two days before onset of cerebral malaria).

Taken together, we showed that OCT and SLO *in vivo* imaging methods enable an early detection of malaria retinopathy in mice and may thus be assessed for early diagnosis of cerebral malaria in human patients.

### Long lasting neurodegenerative effects of malaria in the retina

We found malaria parasites to cause significant neurodegeneration in the retina, especially in parts of the periphery that were associated with blood vessel occlusion. Remarkably, this effect was in part still visible at 18DPI, after 12 days of DHA treatment, corresponding to observation in malaria patients that may suffer from neurological deficits long after overt disease symptoms have subsided^3^. Unfortunately, there are very few longitudinal studies with malaria patients that have addressed this possibility. Yet, a recent study found that children who suffered from cerebral malaria experienced a marked loss of hearing function^4^ suggesting a loss of neuronal cells in the cochlea. Whether a permanent loss of function occurs in the retina (*e*.*g*. loss of visual field) and possibly also in other parts of the CNS, would be an interesting subject for future clinical studies and non-invasive OCT and SLO investigations may be particularly well suited for these.

Parasitaemia was strongly reduced by the DHA treatment, with no parasites detectable in the blood stream after four days. However, even after 12 days of DHA treatment *in vivo* imaging and *ex vivo* histology still showed GFP positive structures in the retina. This raises the question whether these GFP positive parasites have indeed been killed by the treatment and were inert, but not yet removed from the neuronal tissue, or whether they were still viable and capable to induce pathology. The latter case could be explained by parasites being partly shielded from anti-malarial drug treatment inside the neuroretina. If this was the case, novel drug delivery systems developed for the systemic treatment of brain cancer, such as GSH-conjugated, PEGylated liposomes^46,47^, could be adapted to allow anti-malarial drugs to penetrate into the CNS so as to increase on-target drug activity.

The sequestration of parasites in the nervous tissue also induced local inflammation, as evidenced by the GFAP staining. This inflammatory response may in part be responsible for an aggravation of the phenotype even after parasite elimination by DHA treatment. In particular, the aneurysm-like dilations in the retinal vasculature found at 18DPI, but not at 10DPI, may have been due in part to localized inflammatory processes, which in turn also corresponded to local thinning of the retina. Remarkably, we found parasites inside some of the GFAP positive Müller cells suggesting a direct link between parasite neuroretina infiltration and inflammation.

### Malaria parasites compromise the blood retinal barrier

The blood brain barrier (BBB) consists of a highly-specialized network of blood vessels, which provides nutrients and oxygen, and which removes carbon dioxide and metabolic waste (*i*.*e*. urea, creatinine, *etc*.)^48^. The BRB also protects the neuronal tissue from entering of large molecules and pathogens thus ensuring homeostasis of the brain. Although not much is known about the early pivotal pathophysiological changes that lead to cerebral malaria, there is evidence that the pathological mechanisms causing malaria retinopathy and cerebral malaria are the same or very similar^27^.

Furthermore, similar to the brain also the retinal vasculature consists of highly specialized blood vessels, forming the blood-retinal-barrier (BRB). These similarities of the retina to the brain and the accessibility of the eye together with the *in vivo* imaging techniques turns the retina into an ideal model system and valuable tool for the in-depth analysis of pathophysiological mechanisms and to study host - parasite interactions in the central nervous system in malaria disease.

Particularly with respect to an improved management of complications, the verification of the passage of the parasites through the BRB may be of clinical relevance, because, anti-malarial drugs and treatment regimens may have to meet new requirements like *e*.*g*. inhibition of extravasation and retained activity in tissues. In this context, the observation that parasites apparently can use Müller glial cells to penetrate into the neuroretina is especially interesting and conforms to earlier findings on transcellular migration in the brain^49,50^. Our results furthermore indicate that in the retina, malaria parasites can infect Müller glial cells over time, even in the presence of DHA treatment. While, conversely, the parasites not associated with Müller cells (presumably mostly located intravasally) decreased upon treatment, one may speculate that the intracellular localization has a protective effect against systemic antimalarial drugs. This concept may have to be considered in future drug development, especially in the context of cerebral malaria.

## Conclusion

Our work shows that state-of-the-art *in vivo* retinal imaging methods allow for an early detection and follow-up of malaria retinopathy in the mouse. Confirmation of a similar detection advantage in future human patient studies could allow to develop retinal imaging into a clinical tool for the early diagnosis and treatment of cerebral malaria. Our work also opens a new perspective on how malaria parasites may enter the nervous tissue itself. In the retina, Müller glia cells may constitute an important port of entry. Whether the analogous cell type in the brain, the radial glial cell (Bergmann glia in the cerebellum), has a similar role in the pathogenesis of cerebral malaria may be an exciting subject for future studies. The symptoms of malaria retinopathy, as assessed *in vivo* and *ex vivo*, persisted for at least 12 days after the beginning of the DHA anti-malarial treatment. Furthermore, neurodegenerative processes and neuronal cell loss appeared concomitantly with malaria retinopathy. In the mouse model, the combination of *in vivo* imaging techniques with the possibility to extract diseased tissues may prove particularly useful for future investigations into disease mechanisms as well as testing of novel therapeutic approaches.

Taken together, our study suggests that malaria parasites can infiltrate the neuroretina and produce long-lasting neurodegenerative effects. Since, the composition of the BRB and BBB is essentially the same; similar processes are likely to also occur in the brain and lead to long-lasting neurological deficits. This in turn implies that the overall long-term socioeconomic effects of cerebral malaria^51^ may extend well beyond the acute phase of the disease. In future studies on severe human malaria, more emphasis on the identification of sequelae and their prevention shall be laid. This includes sight and hearing^4^, as well as other neurological deficits.

## Supplementary information

Supplemental Video 1

Supplemental Video 2

Supplementary information

## Data Availability

The datasets generated during and/or analysed during the current study (SLO images, OCT scans, ERG files, microscopic images, etc.) are available from the corresponding author on reasonable request.
