# Supplementary material for: A retinal model of cerebral malaria
Source: Sci Rep. 2019 Mar 5;9:3470. doi: 10.1038/s41598-019-39143-z (PMC6401001; doi:10.1038/s41598-019-39143-z)
Supplement: Supplementary file 3 — Supplementary information [file 41598_2019_39143_MOESM3_ESM.pdf]

A retinal model of cerebral malaria

François Paquet-Durand[1&*], Susanne C. Beck[2&], Soumyaparna Das[1], Gesine Huber[2], Le Chang[3,4], Timm Schubert[3,4], Naoyuki Tanimoto[2], Marina Garcia-Garrido[2], Regine Mühlfriedel[2], Sylvia Bolz[3], Wolfgang Hoffmann[5], Ulrich Schraermeyer[6], Benjamin Mordmüller[5], Mathias Seeliger[2]

[1] Cell Death Mechanisms Lab, Institute for Ophthalmic Research, University of Tübingen, Germany.

[2] Division of Ocular Neurodegeneration, Institute for Ophthalmic Research, University of Tübingen, Germany.

[3] Institute for Ophthalmic Research, University of Tübingen, Germany.

[4] Centre for Integrative Neuroscience (CIN), University of Tübingen, Germany.

[5] Institute for Tropical Medicine, University of Tübingen, Germany.

[6] Experimental Vitreoretinal Surgery, Institute for Ophthalmic Research, University of Tübingen, Germany.

* Corresponding Author
E-Mail: francois.paquet-durand@klinikum.uni-tuebingen.de

[&] These authors contributed equally to this work

Supplementary figures and captions

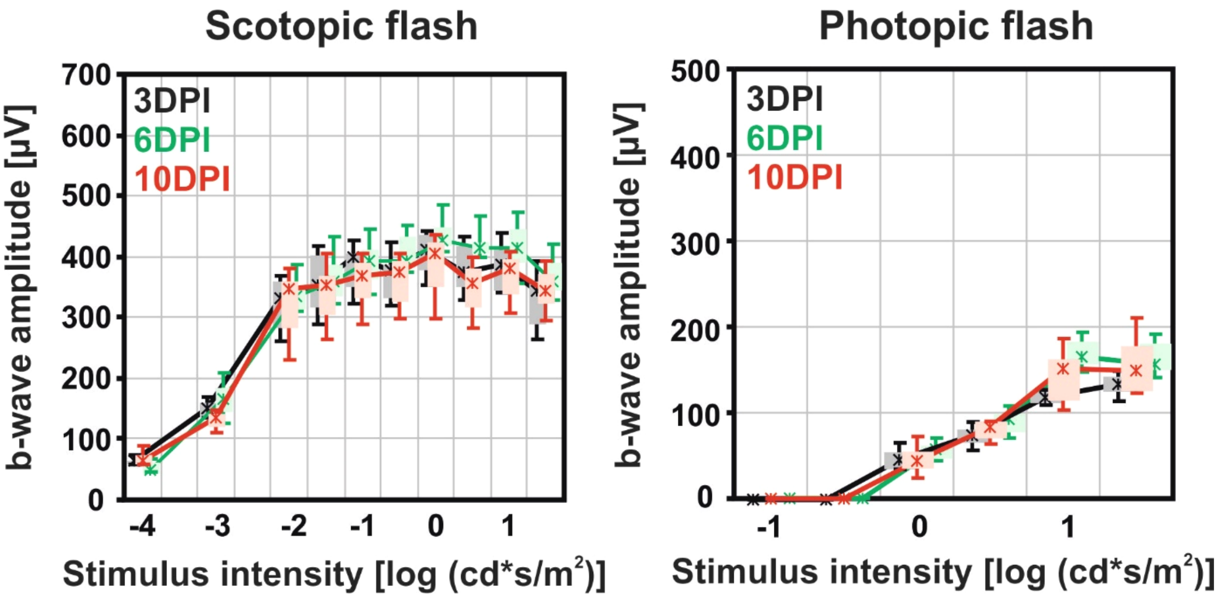

Fig. S1: Functional analysis of the retina during infection. No alterations of the retinal function at 3, 6, and 10DPI (*i.e.* 4 days post treatment), respectively, could be observed in the quantitative evaluation (box-and-whisker plot) of the scotopic (A, left) and photopic (B) b-wave amplitudes (n=4). Boxes indicate 25% and 75% quartiles, whiskers 5% and 95% quantiles and the asterisks the median of the data.

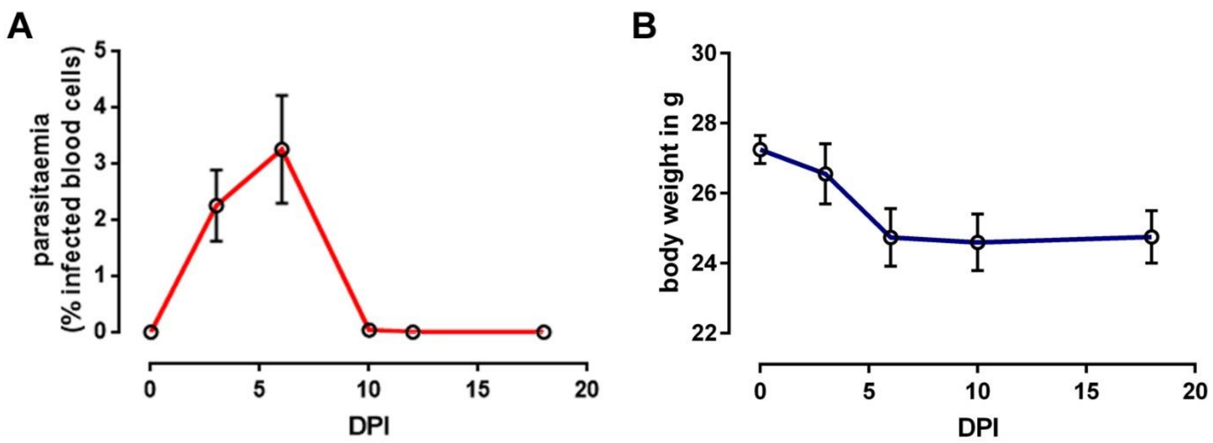

Fig. S2: DHA treatment reduces parasitaemia and prevents further weight loss. (A) After *P. berghei* infection, parasitaemia in the blood stream increased rapidly until the start of the DHA treatment at 6DPI. Afterwards, parasitaemia decreased until at 12DPI no parasites could be detected in blood. (B) The body weight of experimental animals decreased by about 8% until 6DPI. DHA treatment stabilized body weight till the end of the experiment. n = 11 animals (0DPI-6DPI), n = 4-5 animals (10DPI-18DPI).

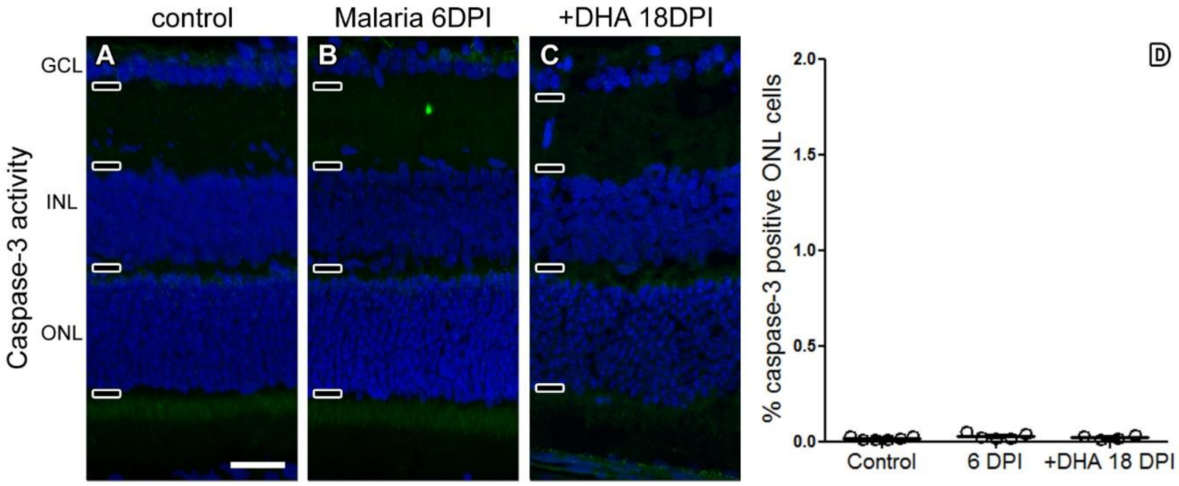

Fig. S3: Cell death in malarial retinopathy is independent of caspase activity. *P. berghei* infected mice presented with significantly increased numbers of dying, TUNEL positive cells at 6 and 18DPI (Figure 6). An immunostaining for activated caspase-3 did, however, not identify any measurable increase of caspase activity at either time-point, indicating that malaria induced neurodegeneration was caspase-independent and non-apoptotic. Scale bar in A = 50 µm; n (control) = 6 observations from 5 animals, n (6DPI) = 6/6; n (18DPI) = 4/4.

Captions for supplemental videos

Supplemental Video 1: Scanning laser ophthalmoscopy (SLO) reveals GFP-labelled parasites *in vivo*. At 3DPI, mice infected with GFP-labelled *P. berghei* presented with fluorescence in non-invasive SLO imaging (*cf*. Fig. 2A). In the video, the GFP-label is visible travelling through the retinal vasculature. Note that the video is repeated twice, first in its original form, then with a red circle highlighting the vessel through which the GFP-label is moving. The video is representative for similar observations made in 11 different infected animals.

Supplemental Video 2: Malaria-induced retinal layer aberrations are visible in optic coherence tomography (OCT). At 3DPI, *P. berghei* infected mice were investigated using non-invasive *in vivo* OCT imaging. The video shows a volume scan through the infected retina superimposed on an SLO image (autofluorescent mode; *cf*. Fig 2A). Consecutive OCT scans are shown, going from inferior (I) towards superior (S), towards the optic nerve head. While large areas of the retina still display normal morphology (*cf.* Fig. 1 OCT), numerous alterations are visible as dark areas of variable size, sometimes leading to strong distortions of retinal layering. Such alterations are observed throughout the entire retina. Note that the video is repeated twice, first in its original form, then with red arrows indicating areas with obvious retinal layer alterations. The video is representative for similar observations made in 11 different infected animals. The volume scan is orientated along the temporo (T) - nasal (N) axis.
